# Treatment of Partial Posterior Cruciate Ligament Injuries with Platelet-Rich Plasma in Growth Factors (PRGF) Intraligamentous Infiltration and a Specific Knee Brace

**DOI:** 10.1055/s-0040-1722342

**Published:** 2021-02-28

**Authors:** David Barastegui, Eduard Alentorn-Geli, Dhaval Gotecha, Marta Rius, Jordi Navarro, Xavier Cuscó, Roberto Seijas, Ramón Cugat

**Affiliations:** 1 Instituto Cugat, Hospital Quirónsalud Barcelona, Barcelona, Spain; 2 Mutualidad Catalana de Futbolistas, Federación Española de Fútbol, Barcelona, Spain; 3 Fundación García-Cugat, Barcelona, Spain; 4 Universitat Internacional de Catalunya, Barcelona, Spain; 5 Pad. Dr. Vithalrao Vikhe Patil Foundation's Medical College Hospital, Ahmednagar, Maharashtra, India

**Keywords:** PCL, treatment, PRP, growth factors, knee brace

## Abstract

Posterior cruciate ligament (PCL) injuries are not as common as other knee ligament injuries, but may present a challenging scenario for even skilled knee surgeons. Complete PCL tears are typically encountered in the setting of multiligament knee injuries and require surgical treatment. Isolated complete PCL injuries are uncommon and the best treatment is debated, and likely depends on the degree of symptoms and objective instability. However, many PCL injuries will be partial tears (grade I or II). The purpose of this chapter is to describe our treatment of choice for partial PCL injuries through a conservative approach.

**Level of evidence** Level IV.

The posterior cruciate ligament (PCL) is the largest and strongest ligament in the knee. PCL injuries are secondary to high-energy trauma such as car accidents or sports injuries. 1 2 This injury typically affects males, with a prevalence ranging from 73 to 97% whether isolated or combined. 1 3

PCL injuries typically present concurrently with other knee injuries, including anterior cruciate ligament (ACL), medial collateral ligament (MCL), or posterolateral corner (PLC) injury. However, isolated injuries of the PCL also play a role in complex posterior injuries of the knee. PCL injuries can be partial (usually referred as grade I or II) or complete (grade III). The majority of grade III PCL tears is associated with multiligament knee injuries, with one study reporting that 79% of these injuries involved the PCL in a trauma setting. 4 Complete PCL injuries were associated with ACL tears in 46% of patients, MCL tear in 31%, and PLC injury in 62% of patients in a trauma setting. 1 2 In their series of PCL reconstructions, Spiridonov et al reported that only 18% were isolated PCL tears. 5

Isolated partial PCL injuries may be treated nonoperatively thanks to its inherent healing potential. 6 7 It should be mentioned although that partial PCL injuries may heal in an elongated, lax, or attenuated morphology. 6 8 9 On the other hand, acute multiligamentary knee injuries with a concomitant or chronic PCL tear are believed to be best treated with surgery. 10 The purpose of this chapter is to describe our conservative treatment strategy to partial isolated PCL injuries.

## Treatment of Partial PCL Injuries

**Fig. 1:**
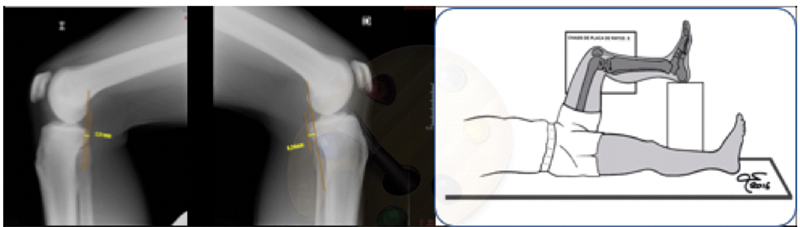
Stress X-rays (gravitational) showing a partial tear of isolated posterior cruciate ligament injury (Courtesy of Dr. Xavier Cuscó).

PCL conservative treatment is a good option in grade I and II isolated injuries (partial tears) provided the following conditions are met:

1. Minimum posterior drawer on physical exam.
2. Less than 10 mm of posterior tibial translation on stress radiographs ( Fig. 1 ).
3. Continuity of PCL fibers on the magnetic resonance imaging (MRI).

Our conservative treatment strategy is based on three main modalities: ultrasound-guided platelet-rich plasma (PRP), use of a specific PCL brace, and early rehabilitation based on quadriceps strengthening.

### Administration of PRP

**Fig. 2:**
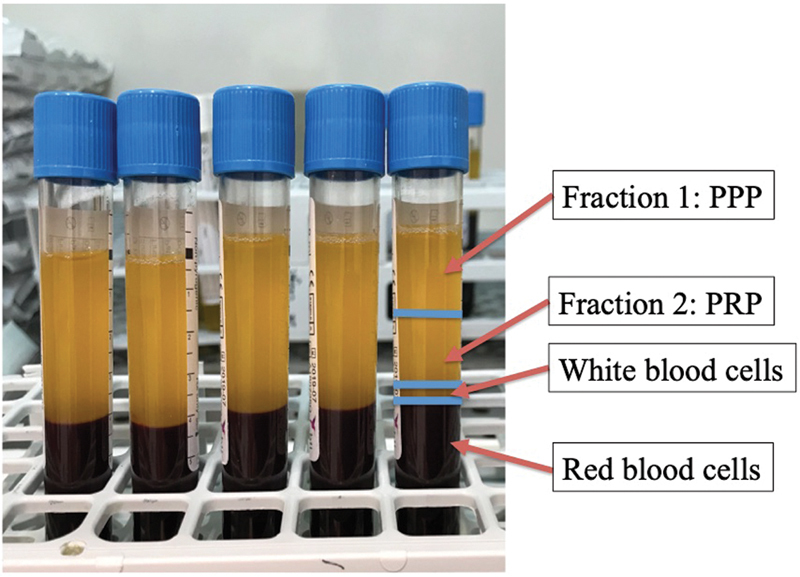
Aspect of blood samples after centrifugation. Note the two fractions of the preparation along with the white and red blood cells layers. PPP, plasma poor in platelets; PRP, plasma rich in platelets.

PRP can be a coadjuvant biological treatment in selected patients to enhance PCL lesion healing. 11 The specific type of PRP that we use is the PRGF Endoret system (BTI Biotechnology Institute, Álava, Spain). Patients come to our clinic fasting for at least 4 hours. Blood samples are extracted using the appropriate tubes with 3.8% citrate solution provided in the kit, which are then placed in a BTI System IV centrifuge machine (BTI Biotechnology Institute, Álava, Spain). Typically, between four and six blood tubes were extracted to obtain 9 or 10-mL tube of plasma. This blood is centrifuged at 1800 rpm for 8 minutes at 580 g. This results in the sedimentation of red and white cells at the bottom and platelets with plasma on the top part of the tubes. 12 The centrifugation process creates two fractions: fraction one is the upper part of the supernatant and is the plasma poor in platelets (PPP); fraction two is the plasma rich in platelets (PRP), which is obtained by extracting the layer just over the white cells ( Fig. 2 ). It is paramount to avoid aspiration of white cells when obtaining fraction two so that no inflammatory reaction is elicited after the injection. The growth factors are activated using CaCl2 at a ratio of 2 IU (international units) per one cc of PRP, maintaining the tubes at room temperature. The activated PPP–PRP at a 50/50 ratio is then injected within the PCL under ultrasound guidance.

**Fig. 3:**
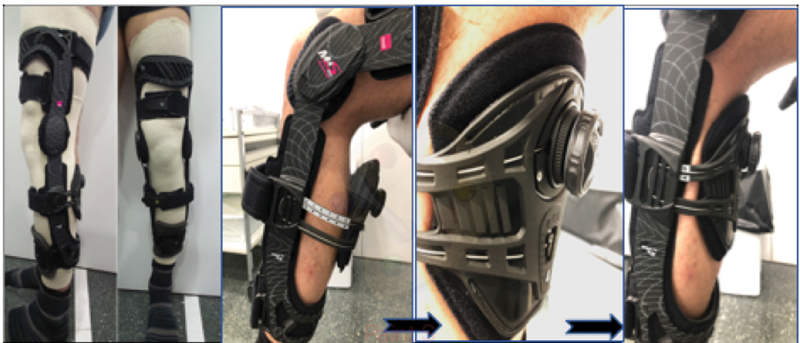
Specific posterior cruciate ligament knee brace from Medi, the M4.s posterior cruciate ligament Dynamic Brace showing the mechanism to counteract posterior tibial sag (Courtesy of Medi Bayreuth Spain SL).

The ultrasound-guided PCL injection allows the permanent visualization of the needle so that one can better control its pathway to the ligament and the exact location where the PRGF is infiltrated ( Fig. 3 ). The best access pathway is the one that will have the shortest and safest trajectory to the ligament with adequate and permanent visualization of the needle and the popliteal neurovascular bundle. For the PCL we use the two planes, the longitudinal or long axis and the transverse or short axis. To better identify and infiltrate the PCL, the patient is placed prone with a bump under the ankles to perform a slight knee flexion. Then, the ultrasound is used to identify the popliteal neurovascular structures and the PCL ( Fig. 3 ). We first begin with the identification of the popliteal fossa in the sagittal plane and the probe is placed in a longitudinal position with respect to the PCL. The ligament runs from the intercondylar part of the medial femoral condyle to the posterior and central part of the lateral tibial plateau. The PCL is seen as a hypoechoic, well-defined structure. We then perform thorough and extensive skin asepsis around the infiltration area. The needle is then advanced parallel to the probe in the long axis in sterile conditions until it reaches the PCL. The exact location of the needle is controlled at all times using both longitudinal axis and transverse axis, and the PRGF is finally administered in the longitudinal axis inside (∼4–5 cc of PRGF) and surrounding the ligament. The remaining part of the PRGF is then administered intraarticularly. The process is repeated two more times typically 2 weeks apart.

### The Specific PCL Brace

**Fig. 4:**
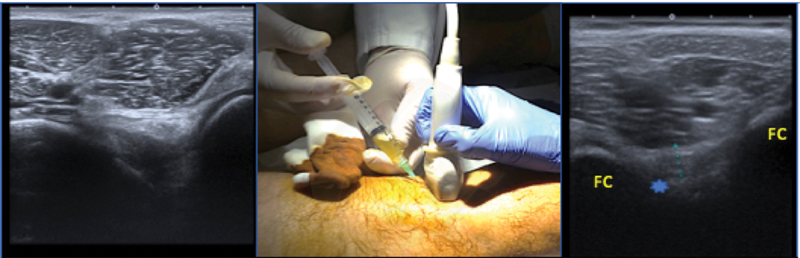
Ultrasound-guided injection of PRGF Endoret (BTI) inside the posterior cruciate ligament sheath (Courtesy of Dr. Marta Rius and Dr. Ramón Cugat). *Determines axial view of posterior cruciate ligament body. FC, femoral condyle.

The patient is placed in the specific M4.s PCL Dynamic Brace (Medi, Bayreuth, Germany) as soon as he/she comes to our clinic with the PCL injury ( Fig. 4 ). The brace applies a constant or dynamic anterior force to counteract posterior sag of the tibia, and therefore helps the healing of the PCL in the right tension. 6 13 The brace hinge should be placed at the level of the femoral condyles, and the straps tightened sequentially following the manufacturer's order. Then, the posterior padded support is tightened to correct the posterior tibial sag. Care should be especially taken to avoid excessive tensioning of the posterior padded support so as to avoid skin injuries. Plain lateral radiographs at 90 degrees of knee flexion are obtained before and after the application of the specific PCL brace to assure that the tension of the brace is adequate to reduce posterior tibial sag. The brace is worn at all times during the first 3 months, and discontinued at rest only during the following month. The patient is evaluated weekly during the first month to assure adequate tolerance and positioning of the brace.

### Rehabilitation Program

The patient undergoes an early rehabilitation program to avoid excessive stiffness and muscular atrophy, while protecting the PCL to assure adequate healing. During the first month, the patient typically performs daily isometric quadriceps strengthening exercises. The patient undergoes weekly gentle mobilization to avoid excessive stiffness, always with the brace on. Range of motion is typically limited to 60 degrees the first month, 120 degrees by the second month, and full motion by the end of the third month. Crutches are recommended during the first 6 to 8 weeks. Progression to dynamic quadriceps strengthening exercises is typically performed at 4 to 6 weeks.

## Results

The results of 13 soccer players with partial PCL injuries (eight grade I and five grade II) treated with the current treatment strategy have been reported. 14 Patients had a mean (range) age of 24.4 (18–29) years, being the right knee in seven and the left knee in six patients. The brace was kept for a mean (range) time of 4.3 (4–5) months. At the end of the treatment, 12 (92.3%) patients achieved a Tegner score of nine and one (7.7%) patient achieved a Tegner score of seven. Evidence of PCL healing on MRI was observed and return to play was achieved in all patients

## Discussion

The principal objective of the present chapter is to evaluate that partial PCL injuries may be treated conservatively through a combination of ultrasound-guided PRGF infiltration to enhance the healing of the ligament, a specific PCL brace to correct the posterior tibial sag and provide the ligament of a better chance to heal in the appropriate tension, and an early rehabilitation program to avoid stiffness and muscle atrophy. However, this treatment protocol requires further clinical research to confirm its usefulness.

The optimization of the treatment and results after PCL injuries is warranted. Some authors have described an increased radiographic progression of osteoarthritis and lower functional outcomes after nonoperative treatment in isolated PCL tears in the long-term. 10 15 Shelbourne et al evaluated isolated PCL tears that were conservatively treated with rehabilitation programs without biological therapies or knee brace and observed radiographic evidence of moderate or severe osteoarthritis in 11% at a mean of 17 years after the injury. 10 Despite the majority of patients reported good subjective outcome scores, incidence of osteoarthritis in middle-aged individuals may have a higher impact later in their life's.

There are limited clinical studies evaluating the outcomes after conservative treatment of PCL tears. Torg et al reported that isolated PCL tears responded favorably to nonoperative treatment at a follow-up of 5.7 years. 16 However, upon further evaluation, the authors found good subjective functional scores and a healed appearance of the PCL on MRI at short-term follow-up (1.7 and 2.6 years) after isolated PCL injury, but less than satisfactory objective scores. 16 As a result, these authors concluded that the PCL treated nonoperatively likely healed with laxity and led to poor objective outcomes.

The effectiveness of PRP is still controversial due to insufficient literature or lack of consensus on the clinical outcomes, likely related to the high heterogeneity of PRP preparation methods used leading to very different products to be applied. 17 18 Studies demonstrating a clear benefit on the application of PRP or PRGF to treat PCL injuries are missing. However, some studies have concluded that PRP can enhance healing in partial ACL injuries, 19 20 21 and may increase the maturation process of postoperative ACL grafts. 22 23 24 Because the PCL is histologically similar to the ACL, it might be argued that the treatment with PRP/PRGF infiltrations can enhance the healing process of partial PCL tears. Anatomically speaking, the PCL is fully covered by synovial tissue, so intra-articular injections of PRP/PRGF may not reach the ligamentous tissue in cases where this membrane is not severely damaged. Therefore, ultrasound-guided local infiltration of PRGF completely inside the synovial sheath is a better choice to assure that the PRP reaches the damaged tissue ( Fig. 3 ).

There is limited evidence on the effectiveness of a specific PCL knee brace to allow adequate ligament healing. Jacobi et al reported that the use of a dynamic PCL brace for 4 months after an isolated acute PCL tear significantly reduced the initial posterior sag of 7.1 mm to 2.3 and 3.2 mm at 12 and 24 months, respectively. 6 On MRI, the PCL was in continuity in 95% of the patients at 6 months. Moreover, the Lysholm score did not show a statistically significant difference between the preinjury period and the 12- and 24-month postinjury periods. 6 The absence of significant differences is a desired finding, as the patients were able to return to the preinjury functionality.

## Conclusions

A combination of ultrasound-guided PRGF infiltration to enhance the healing of the ligament, a specific PCL brace to correct the posterior tibial sag and provide the ligament of a better chance to heal in the appropriate tension, and an early rehabilitation program to avoid stiffness and muscle atrophy may be considered as a treatment option for conservative partial PCL injuries. This treatment modality was effective to achieve adequate MRI-based healing in 100% and a return to play in 90% of soccer players.
